# Transcranial Brain Parenchyma Sonographic Findings in Familial and Sporadic Amyotrophic Lateral Sclerosis

**DOI:** 10.1111/ene.70272

**Published:** 2025-07-13

**Authors:** Ivo Bozovic, Emir Licina, Bogdan Bjelica, Ognjen Milicevic, Aleksa Palibrk, Ivana Basta, Stojan Peric, Aleksandra Pavlovic, Zorica Stevic, Milija Mijajlovic

**Affiliations:** ^1^ Neurology Clinic University Clinical Center of Serbia Belgrade Serbia; ^2^ Faculty of Medicine University of Belgrade Belgrade Serbia; ^3^ Department of Neurology Hannover Medical School Hannover Germany; ^4^ Faculty of Special Education and Rehabilitation University of Belgrade Belgrade Serbia

**Keywords:** amyotrophic lateral sclerosis, familial ALS, nucleus raphe, substantia nigra, transcranial sonography

## Abstract

**Background:**

Amyotrophic lateral sclerosis (ALS) is a progressive neurodegenerative disorder affecting motor neurons. Transcranial sonography (TCS) is a valuable tool for assessing deep brain structures. This study aimed to analyze TCS findings in both sporadic (sALS) and familial ALS (fALS) patients and compare them to healthy controls (HC).

**Methods:**

This cross‐sectional study included 278 patients with sALS and 31 patients with genetically confirmed fALS, and 93 age‐ and gender‐ matched HC. TCS was used to assess substantia nigra (SN) and brainstem raphe (BR) echogenicity and third ventricle diameter (TVD). Functional disability was evaluated using the ALS Functional Rating Scale‐Revised.

**Results:**

BR hypoechogenicity was more frequent in fALS (41.9%) and sALS (37.4%) patients, compared to HC (10.8%) (*p* < 0.001). Right SN hyperechogenicity was observed in 28.1% of sALS, 16.1% of fALS, and 8.6% of HC (*p* = 0.004). Left SN hyperechogenicity was found in 33.5% of sALS, 29.0% of fALS, and 4.3% of HC (*p* = 0.004). SN hyperechogenicity findings on either side were highest in sALS (48.4%) compared to fALS (31.0%) and HC (13.3%) (*p* < 0.001), with a borderline difference between fALS and sALS (*p* = 0.08). BR hypoechogenicity and SN hyperechogenicity were more common in male patients. Increased TVD correlated with older age, later disease onset, bulbar onset, and lower MMSE scores.

**Conclusions:**

TCS is an easily applicable and sensitive diagnostic tool that offers novel insights into several brainstem structures and identify significant differences in their echogenicity between ALS patients and healthy controls, while pointing out similar but not identical patterns of echogenicity in both ALS forms.

## Graphical abstract

This study aimed to analyze transcranial brain parenchyma sonographic (TCS) findings in both sporadic (sALS) and familial amyotrophic lateral sclerosis (fALS) patients and compare them to healthy controls (HC). TCS is a practical and sensitive diagnostic tool that provides novel insights into brainstem structures, highlighting significant echogenicity differences between ALS patients and healthy controls while revealing similar yet distinct patterns in sporadic and familial ALS.

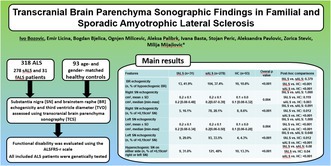

## 1 Introduction

Amyotrophic lateral sclerosis (ALS) is a progressive neurodegenerative disorder which primarily affects motor neurons of the cerebral motor cortex (upper motor neuron, UMN), brain stem and the spinal cord (lower motor neuron, LMN), resulting in weakness and atrophy of limb, bulbar and/or respiratory muscles [1]. Other main clinical variants of motor neuron disease (MND) include primary lateral sclerosis (PLS) with mainly UMN involvement, progressive muscular atrophy (PMA) with dominant LMN neuron degeneration and progressive bulbar atrophy (PBA), a rare and pure brainstem variant. These motor neuron diseases are quite less common compared to typical ALS, and most of these patients usually evolve over time into a typical form with both upper and lower motor neuron signs [2]. Although sporadic ALS (sALS) represents the majority of ALS patients (90%–95%), a clear family history is present in up to 5%–10% of all ALS cases. The inheritance in these patients is mostly autosomal dominant, with the most frequent mutations being in the *C9orf72* and *SOD1* genes [3, 4]. Although patients with familial ALS (fALS) usually have similar clinical presentation as the sporadic form, they also tend to have several specific clinical and epidemiological features [5].

Transcranial brain parenchyma sonography (TCS) is a non‐invasive method of visualization of different subcortical and brainstem structures such as basal ganglia and substantia nigra, which have a proven pathophysiological and diagnostic significance in different neurodegenerative disorders [6]. This relatively low‐cost method has been previously studied as a supplementary imaging tool in several neurological disorders, such as Parkinson's disease (PD) [7], myotonic dystrophies, as well as in ALS [8]. The rationale for employing TCS in ALS diagnostics is based on other neuroimaging modality findings of progressive brainstem pathology, basal ganglia volume reduction, and abnormal striatal dopaminergic function [9, 10, 11, 12, 13, 14]. Although magnetic resonance imaging (MRI) is still considered the gold standard for neuroanatomical visualization, TCS offers unique advantages in ALS, including real‐time bedside assessment (particularly valuable for patients with severe functional disability) [8], positive cost‐effectiveness ratio—especially in low‐income countries (TCS costs typically do not exceed more than 20% of MRI costs) [7], and absence of contraindications for serial monitoring (e.g., in patients with significant respiratory compromise or claustrophobia) [6, 8]. However, a few previous studies of TCS in ALS were mostly focused on sporadic forms of ALS and had a smaller cohort of patients [12, 13]. To the best of our knowledge, this study is the first one to evaluate different TCS findings in a large number of ALS patients, respecting both familial and sporadic forms of this rare disease.

Thus, the aim of this study was to analyze different TCS features and their clinical relevance in a large cohort of ALS patients (including both familial and sporadic ALS patients) and to compare these findings to healthy controls (HC).

## 2 Materials and Methods

### 2.1 Patients' Data

This retrospective cross‐sectional study comprises 318 patients and 93 healthy control subjects. In detail, complete medical histories of 900 ALS patients from the In‐patient clinic of the Neurology Clinic of the University Clinical Center of Serbia were reviewed from 2013 to 2024. After applying the exclusion criteria (insufficient temporal bone window, final diagnosis other than MND, presence of percutaneous endoscopic gastrostomy invasive/non‐invasive mechanical ventilation dependence), the final analysis included a total number of 318 patients with a diagnosis of MND and who had undergone TCS. Our research also included 93 age‐ and gender‐matched healthy controls (HC) who had no neurological, psychiatric, or somatic diseases. The Local Ethics Committee of the Neurology Clinic has approved this study, and it was conducted according to the Declaration of Helsinki. We have obtained signed informed consent from all participants before blood sampling, ultrasonography examination and appliance of different questionnaires/measures.

### 2.2 Clinical Assessment

All patients fulfilled the diagnostic El Escorial revised criteria for definite or probable ALS [15]. Different sociodemographic, clinical, and diagnostic data of all included ALS patients were collected from patients' medical history. Data from the Mini Mental State Examination (MMSE) test results were collected from patients' medical records as available, together with data about the presence of clinical signs of cognitive impairment (including dementia) and depressive symptoms. Neuropsychological examination was conducted in all patients who were suspected to have a cognitive decline. The revised ALS Functional Rating Scale (ALSFRS‐R) was evaluated in all patients in order to assess their functional status. ALSFRS‐R is a disease‐specific severity measure which clearly reflects disability progression in ALS patients. It comprises 12 items graded with five levels of severity (range from most severe disability—score 0 to the mildest disability—score 4, total score ranging from 0 to 48) [16]. Baseline progression rate was calculated using the formula (48—ALSFRS‐R)/disease duration, with median values of 0.19 (0.07, 0.63) in the FALS group and 0.67 (0.25, 1.00) in the sporadic ALS group (*p* < 0.001).

### 2.3 Genetic Testing

All included ALS patients were genetically tested for ALS‐associated genetic mutations. Patients were classified under fALS if they had a confirmed genetic mutation and/or a positive family history. For fALS patients, the type of mutation was noted as available. Peripheral blood was collected from all patients, and genomic DNA was isolated using standard protocols in the genetics laboratory of the University Neurology Clinic. PureLink Genomic DNA Mini Kit (Life Technologies, USA) was used for DNA extraction from peripheral blood, following the manufacturer's protocol. For the Southern blot method, DNA was salted out using a modified method as previously reported [17]. All coding regions of *SOD1* and *ANG* genes, and several regions of *TDP‐43* (exon 6) and *FUS* (exons 15 and 16) genes were initially amplified and directly sequenced by the Sanger method. A specific two‐step protocol and fragment analysis were performed as previously described, in order to detect the hexanucleotide repeat expansion in the *C9orf72* gene [18, 19]. Whole exome sequencing (WES) was further used to evaluate initially negative genetic results in the 3billion laboratory in Seoul, South Korea.

### 2.4 Transcranial Brain Parenchyma Sonography

Ultrasonography examinations were performed at the Neurology Clinic UKCS using Aloka Prosound Alpha 10 (Aloka, Japan) ultrasound system. The examination was conducted according to the current guidelines [7, 20] and evaluated by a TCS expert who was *naïve* to relevant patients' clinical and genetic data. We have assessed the echogenicity of the substantia nigra (SN) and brainstem raphe (BR) and the diameter of the third ventricle (DTV) in concordance with current methodological guidelines [21]. Axial TCS measured SN hyperechogenic region according to current criteria (values below 0.19 cm^2^ are normal, whereas those greater than 0.19 cm^2^ were deemed hyperechogenic) [6, 7]. A two level‐grading system was implemented for BR evaluation. Grade 0 signifies discontinuous or missing BR, while grade 1 indicates normal, hyperechogenic continuous BR [7]. The third ventricle width was measured in a standardized axial scanning plane of the diencephalon, defined by the smallest transverse diameter on an axial TCS scan [7, 8]. A width of less than 7 mm in patients under 60 years old, or less than 10 mm in those aged 60 and older, was considered normal [6].

### 2.5 Statistical Analysis

Statistical analyses were performed using IBM SPSS Statistics version 29 (Chicago, IL, USA). Data normality was assessed using the Shapiro–Wilk and Kolmogorov–Smirnov tests, complemented by visual inspection through quantile–quantile (Q‐Q) plots. Depending on the data distribution, group differences in continuous variables were analyzed using either the Mann–Whitney *U* test or an independent samples *t*‐test. Associations between categorical variables were assessed using the chi‐squared test or Fisher's exact test, as appropriate. Bivariate correlations were analyzed using Pearson's correlation coefficient for normally distributed data and Spearman's rank correlation coefficient for non‐normally distributed data. Receiver operating characteristic (ROC) curve analyses were conducted to evaluate the diagnostic performance of SN hyperechogenicity in distinguishing ALS patients from HC. The area under the ROC curve (AUC) was calculated, with AUC values > 0.9 indicating excellent performance and > 0.8 indicating good performance. Optimal cutoff values for SN hyperechogenicity were determined using Youden's index and closest‐to‐top‐left analysis, providing corresponding sensitivity and specificity. A two‐tailed *p* < 0.05 was considered statistically significant for all analyses.

## 3 Results

### 3.1 Study Cohort

The study cohort included 278 patients with sALS, 31 patients with fALS, and 93 age‐ and gender‐matched healthy controls. Main sociodemographic and clinical features of all ALS patients and healthy controls are presented in Table 1. There were no statistically significant differences in gender and age at testing between the groups. Patients with fALS had an earlier disease onset (53.8 ± 12.0 vs. 58.7 ± 11.0 years, *p* = 0.021), less frequent cognitive disturbances (3.2% vs. 16.3%, *p* = 0.035), and higher ALSFRS‐R score (ALSFRS‐R total score mean: 40.8 ± 5.3 vs. 38.1 ± 6.4, *p* = 0.038) compared to patients with sALS.

**TABLE 1:** Main sociodemographic and clinical features of all ALS patients and healthy controls.

Features	fALS (*n* = 31)	sALS (*n* = 278)	HC (*n* = 93)	*p*
Male gender, mean (%)	14 (45.2%)	152 (53.2%)	48 (51.6%)	0.565
Age at testing in years, mean ± SD	57.6 ± 11.8	60.0 ± 10.5	59.4 ± 13.9	0.650
Age at onset in years, mean ± SD	53.8 ± 12.0	58.7 ± 11.0	—	0.021
Disease duration in years, mean ± SD	3.84 ± 3.79	1.35 ± 2.47	—	< 0.001
Disease onset: spinal, *n* (%)	29 (93.5%)	201 (72.3%)	—	< 0.001
Disease onset: bulbar, *n* (%)	2 (6.5%)	77 (27.7%)	—	< 0.001
Family history *n* (%)	23 (74.2%)	0 (0.0%)	—	< 0.001
Gene mutation, *n* (% of all fALS patients)	*C9orf72*: 4 (12.9%); *SOD1*: 23 (74.2%); Other: 4 (12.9%)	—	—	—
Subjective cognitive disturbance *n*, (%)	1 (3.2%)	45 (16.3%)	—	0.035
Dementia, *n* (%)	0 (0.0%)	7 (3.7%)	—	0.475
MMSE, mean ± SD	28.0 ± 2.3	26.8 ± 3.2	—	0.344
Depressive symptoms *n* (%)	4 (12.9%)	28 (10.1%)	—	0.406
ALSFRS‐R total score, mean ± SD	40.8 ± 5.3	38.1 ± 6.4	—	0.038
Baseline progression rate Median (P25, P75)	0.19 (0.07, 0.63)	0.67 (0.25, 1.00)	—	< 0.001

Abbreviations: ALSFRS‐R, ALS Functional Rating Scale–Revised; *c9orf72*, chromosome 9 open reading frame 72; fALS, familial amyotrophic lateral sclerosis; HC, healthy controls; MMSE, mini‐mental state examination; sALS, sporadic amyotrophic lateral sclerosis; SD, standard deviation; *SOD1*, superoxide dismutase 1.

### 3.2 Main TCS Findings and Comparison Between Groups

Main transcranial sonography findings in both cohorts of our ALS patients and HC are shown in Table 2.

**TABLE 2:** Transcranial sonography findings in ALS patients and healthy controls.

Features	fALS (*n* = 31)	sALS (*n* = 278)	HC (*n* = 93)	Overall *p*	Post‐hoc comparisons
DVT mm, mean ± SD mm, median [min‐max]	5.7 ± 1.6 5.0 [3.0–10.0]	6.5 ± 2.2 6.0 [3.0–15.0]	6.1 ± 2.2 6.0 [3.0–13.0]	0.068	—
Increased DVT (*n*, % of > 7 mm)	3, 9.7%	75, 27.0%	19, 20.4%	0.065	—
BR echogenicity (*n*, % of hypoechogenic BR)	13, 41.9%	104, 37.4%	10, 10.8%	< 0.001	fALS versus sALS: 0.379 fALS versus HC: < 0.001 sALS versus HC: < 0.001
Right SN echogenicity cm^2^, mean ± SD cm^2^, median [min–max]	0.2 ± 0.1 0.2 [0.08–0.48]	0.2 ± 0.1 0.2 [0.07–0.39]	0.1 ± 0.0 0.1 [0.08–0.32]	0.004	fALS versus sALS: 1.000 fALS versus HC: 0.119 sALS versus HC: 0.003
Right SN echogenicity (*n*, % of > 0,19 cm^2^ SN)	5, 16.1%	78, 28.1%	8, 8.6%	< 0.001	fALS versus sALS: 0.091 fALS versus HC: 0.012 sALS versus HC: < 0.001
Left SN echogenicity cm^2^, mean ± SD cm^2^, median [min–max]	0.2 ± 0.1 0.2 [0.08–0.34]	0.2 ± 0.1 0.2 [0.06–0.50]	0.1 ± 0.0 0.1 [0.06–0.28]	0.004	fALS versus sALS: 1.000 fALS versus HC: < 0.001 sALS versus HC: < 0.001
Left SN echogenicity (*n*, % of > 0,19 cm^2^ SN)	9, 29.0%	93, 33.5%	4, 4.3%	< 0.001	fALS versus sALS: 0.648 fALS versus HC: 0.012 sALS versus HC: < 0.001
Hyperechogenic SN on either side (*n*, % of > 0,19 cm^2^ right or left SN)	9, 31.0%	121, 48%	10, 13.3%	< 0.001	fALS versus sALS: 0.08 fALS versus HC: 0.04 sALS versus HC: < 0.001

*Note:* cm^2^, echogenic area size.

Abbreviations: BR, brainstem raphe; DVT, third ventricle diameter; fALS, Familial amyotrophic lateral sclerosis; HC, healthy controls; sALS, sporadic amyotrophic lateral sclerosis; SD, standard deviation; SN, substantia nigra.

#### 3.2.1 Brainstem Echogenicity

**GRAPH 1:**
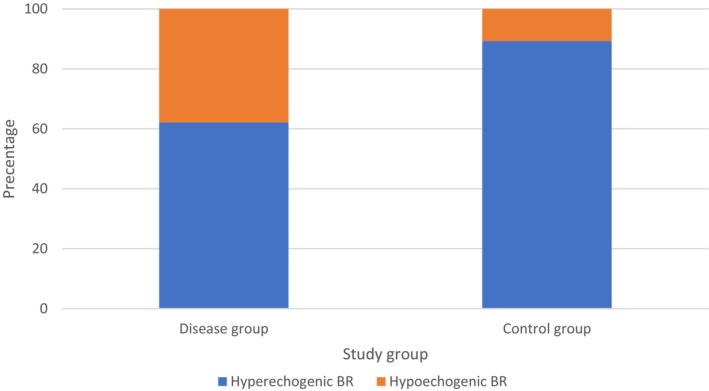
Proportions of hypoechogenic brainstem raphe (BR) in disease versus control groups.

Significant differences were observed in the proportion of participants with hypoechogenic brainstem raphe (*p* < 0.001). The fALS group had 41.9% of participants with hypoechogenic BR, which was comparable to 37.4% in the sALS group (*p* = 0.379); both groups exhibited significantly higher proportions compared to controls (10.8%) (fALS vs. HC: *p* < 0.001, sALS vs. HC: *p* < 0.001) (Graph 1).

#### 3.2.2 Right Substantia Nigra Echogenicity

**GRAPH 2:**
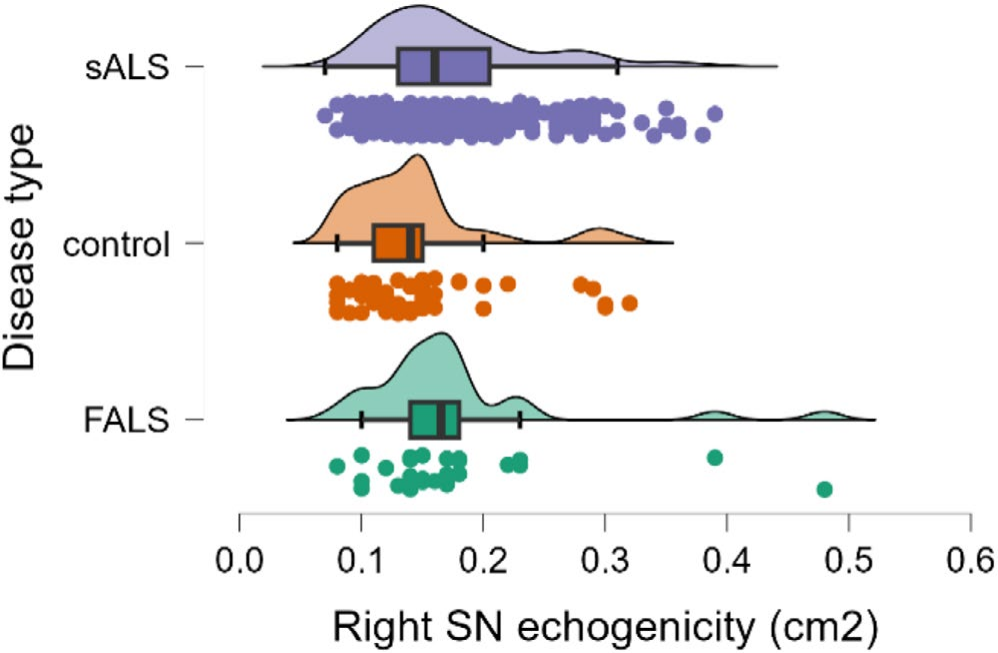
Raincloud plots of right substantia nigra echogenicity in sALS, FALS, and HC.

The right SN echogenicity showed differences between groups (*p* = 0.004). The mean echogenicity was 0.2 ± 0.1 cm^2^ for both fALS and sALS, and 0.1 ± 0.0 cm^2^ for HC; however, the difference was statistically significant only between sALS and HC (sALS vs. HC: *p* = 0.003). Hyperechogenicity of the right SN was observed in 28.1% of sALS participants, significantly higher than the 8.6% observed in HC (*p* < 0.001). The results in fALS participants showed right SN hyperechogenicity in 16.1%, which was also greater than HC (*p* = 0.012) but not significantly different from sALS (*p* = 0.091) (Graph 2). A cut‐off value of 0.155 cm^2^ for right SN hyperechogenicity distinguished ALS patients from HC with a sensitivity of 55.7% and a specificity of 77.6% (AUC 0.67, 95% CI [0.59–10.75]).

#### 3.2.3 Left Substantia Nigra Echogenicity

**GRAPH 3:**
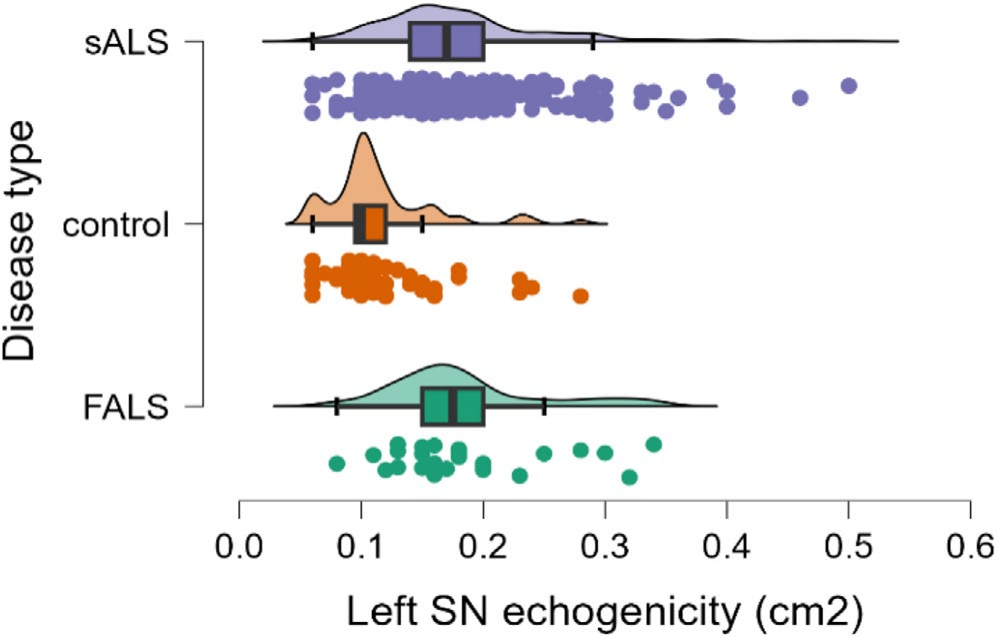
Raincloud plots of left substantia nigra echogenicity in sALS, FALS, and HC.

Left SN echogenicity also showed significant differences between groups (*p* = 0.004). The mean echogenicity was 0.2 ± 0.1 cm^2^ in both fALS and sALS, and significantly greater compared to 0.1 ± 0.0 in HC (fALS vs. HC: *p* < 0.001, sALS vs. HC: *p* < 0.001). No significant difference was observed between fALS and sALS. Hyperechogenicity of the left SN was observed in 29.0% of fALS and 33.5% of sALS patients, and 4.3% of HC, with significant differences between HC and both fALS (*p* = 0.012) and sALS (*p* < 0.001), but not between fALS and sALS (*p* = 0.648) (Graph 3). For left SN hyperechogenicity, a cut‐off value of 0.125 cm^2^ distinguished ALS patients from HC with a sensitivity of 82.5% and specificity of 76.1% (AUC 0.83, 95% CI [0.77–0.89]).

#### 3.2.4 Combined Substantia Nigra Hyperechogenicity

Participants with SN hyperechogenicity on either side were more frequent in sALS (48.4%) compared to fALS (31.0%) and HC (13.3%), with significant overall group differences (*p* < 0.001). Post hoc comparisons revealed a significant difference between fALS and HC (*p* = 0.04) and between sALS and HC (*p* < 0.001), while the difference between fALS and sALS was of borderline significance (*p* = 0.08).

#### 3.2.5 Third Ventricle Diameter

The third ventricle diameter (DVT) did not differ significantly between the groups (*p* = 0.068). The mean DVT was 5.7 ± 1.6 in the fALS group, 6.5 ± 2.2 mm in the sporadic ALS group, and 6.1 ± 2.2 mm in healthy controls. The median values were similar across groups, with 5.0 mm for fALS and 6.0 mm for both sALS and HC. Additionally, the proportion of participants with an enlarged DVT (greater than 7 mm) was highest in the sALS group (27.0%), followed by HC (20.4%) and fALS (9.7%), though the differences were not statistically significant (*p* = 0.065).

#### 3.2.6 Correlation of the TCS Findings With Sociodemographic and Clinical Features in All ALS Patients

We found no significant differences in age at testing, age at onset, disease onset site, positive family history, cognitive disturbances, depressive symptoms, dementia, MMSE scores, or ALSFRS‐R total scores between patients with and without BR hypoechogenicity (*p* > 0.05). However, BR hypoechogenicity was more prevalent among male patients (59.9% vs. 43.9%, *p* = 0.004).

Similarly, no significant differences were observed in the same variables between patients with and without right SN hyperechogenicity (*p* > 0.05), although right SN hyperechogenicity was more common in males (68.7% vs. 46.5%, *p* = 0.004).

For left SN hyperechogenicity, there were also no significant differences in age at testing, age at onset, disease onset site, positive family history, cognitive disturbances, depression, dementia, MMSE scores, or ALSFRS‐R total scores (*p* > 0.05). Left SN hyperechogenicity, however, was significantly more frequent in male patients (71.6% vs. 44.0%, *p* < 0.001).

Patients with increased DVT were significantly older (65.2 ± 9.0 vs. 57.9 ± 10.5 years, *p* < 0.001), had a later disease onset (63.8 ± 9.5 vs. 56.3 ± 11.5 years, *p* < 0.001), and lower MMSE scores (25.9 ± 3.9 vs. 27.3 ± 2.7, *p* = 0.013). They also more frequently had bulbar onset disease (35.9% vs. 22.1%, *p* = 0.013) compared to patients with normal DVT. No differences were observed in gender, ALSFRS‐R total score, presence of cognitive disturbances, depressive symptoms, or presence of dementia.

## 4 Discussion

This study aimed to analyze different TCS findings and their clinical relevance in a large cohort of ALS patients and to compare these findings to HC. To the best of our knowledge, this is the first study which directly evaluated TCS findings in FALS patients.

We have noted that BR hypoechogenicity (grade 0) was more frequent in both fALS and sALS patients, compared to HC. These findings are similar to our previous data where BR hypoechogenicity was observed from one third to up to half of patients with other neuromuscular disorders such as myotonic dystrophies [20, 22]. BR hypoechogenicity has been typically associated with depressive symptoms in a number of other neurological diseases, such as PD, restless leg syndrome, Huntington's disease, epilepsy and migraine [23, 24]. It is postulated that a reduced echogenic BR signal in TCS reflects the disruption of the serotonergic tracts in the dorsal nucleus raphe, which might be a prerequisite for the occurrence of depression in these patients [20, 25, 26, 27, 28]. In addition, a marked reduction of serotonergic receptor binding was observed in post‐mortem histopathologic brain tissue studies of patients with ALS [29]. Although it is already known that the prevalence of depressive symptoms rises with older age [30], BR hypoechogenicity was still more frequent in our fALS (younger in average) compared to sALS (older in average) patients, however we have not found a statistically significant difference in depressive symptoms between fALS and sALS patients (probably due to small fALS sample size). Thus, TCS should be considered as a reliable adjuvant tool for screening and/or diagnosing depression in all ALS patients and might justify an early serotonin reuptake inhibitors (SSRI) therapy initiation in these patients.

Increased SN echogenic size (hyperechogenicity) is a characteristic TCS finding in patients with different neurodegenerative disorders, such as Parkinson's disease, and might be a result of iron accumulation in several brain regions [7]. On the other hand, increasing evidence implies that the dysfunction of iron metabolism is probably involved in both onset and progression of ALS [31]. Thus, it might be postulated that different types of histopathologically confirmed brain accumulations, already proven in ALS patients, could consequentially lead to impaired iron metabolism in these patients. These findings are in accordance with our results where SN hyperechogenicity on either side was observed in at least one third of both cohorts of ALS patients, although slightly more frequent in patients with sALS. Moreover, German authors have also noted that SN hyperechogenicity was present in up to half their ALS patients [32]. Both Serbian and German results are significantly higher compared to general population data, where around 10% of healthy persons had SN hyperechogenicity [20]. These TCS findings in ALS patients could be at least a partial explanation for the occasional presence of cardinal parkinsonism signs (without complete PD diagnostic criteria) in these patients and further accelerate dopaminergic treatment initiation as needed.

Our findings indicate that SN hyperechogenicity was more commonly found in male compared to female patients. This data is in accordance with previous literature data about other neurodegenerative diseases (such as PD), where increased SN echogenicity was more frequently associated with male gender [32]. It is suspected that male patients with neurodegenerative disorders might have a greater iron accumulation and lower neuromelanin levels, but hormonal (especially estrogen) or even genetic influence should not be underestimated [33, 34, 35, 36]. On the other hand, to the best of our knowledge, no research in to‐date literature has reported gender differences in BR hypoechogenicity evaluated by TCS in ALS patients. However, the influence of hormones on the brain's serotonergic system may play a role in these patients, though it remains insufficiently understood.

There was no difference in mean DVT among groups and, although the proportion of patients with enlarged third ventricle findings was greater in sALS cohort, it did not reach statistical significance. This is in conflict with a previous study where the DVT was markedly increased in ALS patients compared to controls [8]. An enlarged third ventricle was more commonly noted in older ALS patients, with bulbar onset and lower MMSE results. Although this finding could be a result of normal aging and natural brain mass reduction, it can be speculated that DVT enlargement may be more frequently observed in ALS patients with cognitive decline, a non‐motor manifestation in at least 15% and up to 65% of ALS patients (more frequently observed in patients with bulbar onset) [37, 38]. Thus, neurologists should pay more attention to these patients and provide further neuropsychological testing as needed.

Finally, while respecting the fact that modern MRI techniques provide superior neuroanatomical spatial resolution, our findings still underline the role of TCS as a complementary neuroimaging tool in ALS. For instance, TCS SN hyperechogenicity in patients with neurodegenerative disorders may reflect (or even precede) iron accumulation detectable by susceptibility‐weighted imaging (SWI) on MRI [7, 14]. On the other hand, TCS captures these changes at the bedside without significant costs, patient transportation and limiting contraindications, compared to MRI [6, 7, 8]. This is especially critical for functionally disabled ALS patients or ALS patients where severe bulbar or respiratory dysfunction might complicate adherence to MRI. Moreover, one might speculate that different TCS echogenicity alterations (e.g., BR hypoechogenicity) could reflect early serotonergic dysfunction before MRI‐visible atrophy [25, 29] and might even serve as a potential prodromal biomarker for ALS. However, additional research combining TCS and MRI findings is still needed in order to clarify their synergistic roles in ALS.

Over the past few years, there has been a growing number of studies based on the identification of different potential biomarkers in ALS. However, most of these biomarkers are fluid‐based (such as neurofilament proteins, circulating ribonucleic acids (RNAs), oxidative stress and neuroinflammation markers etc.) and usage of fluids as a source of biomarkers often comes with a set of specific and well‐known difficulties [38, 39, 40, 41, 42, 43]. On the other hand, although several risk factors for cognitive impairment appearance in ALS patients (like the presence of *C9orf72* repeat expansion, positive ALS family history, bulbar disease onset etc.) are well known, the TCS related predictors of cognitive decline in these patients are still underexplored among current literature data [44, 45]. However, a recent Chinese study has shown that enlarged DVT might predict cognitive decline in another neurodegenerative disorder, such as PD [46]. These findings are in accordance with our study where increased DVT was more frequently observed in ALS patients with cognitive impairment. Thus, TCS as a biomarker might offer a real‐time, continuous and non‐invasive window into structural and predict cognitive brain changes in ALS patients. Nevertheless, this ultrasonographic method as a biomarker is still understudied in ALS and future studies are needed in order to validate TCS findings against fluid, cognitive and other biomarkers in both familial and sporadic forms of ALS. *Limitations*.

The main limitations of this study are the lack of a prospective approach and its cross‐sectional design. Another limitation of the study is the relatively small number of fALS patients and the lack of differentiation of these patients based on specific genetic mutations. Moreover, concurrent MRI findings as well as additional upper motor neuron‐specific assessments (e.g., Penn Upper Motor Neuron Score) were not included in our research, and future correlative studies are needed in order to align different TCS echogenicity patterns with these measures. Nevertheless, our findings still highlight the clinical relevance of TCS in ALS, complementing existing MRI approaches. Another limitation of our study is that we have not used standardized diagnostic criteria for depression in all our patients (we have conducted relevant data from patients' medical records). However, to the best of our knowledge, this is the first study to analyze different TCS findings in both genetically confirmed familial and sporadic ALS patients, further compared to age‐ and gender‐matched HC.

## 5 Conclusions

BR hypoechogenicity and SN hyperechogenicity were commonly noted in both fALS and sALS patients, compared to HC. Older ALS patients with bulbar disease onset and cognitive decline were more likely to have enlarged DVT. TCS is a practical and sensitive diagnostic tool that provides novel insights into brainstem structures, highlighting significant echogenicity differences between ALS patients and healthy controls while revealing similar yet distinct patterns in sporadic and familial ALS.

## Author Contributions

**Ivo Bozovic:** conceptualization, investigation, methodology, validation, visualization, supervision, writing – review and editing, writing – original draft. **Emir Licina:** conceptualization, data curation, writing – review and editing, writing – original draft, investigation. **Bogdan Bjelica:** writing – original draft, methodology, software, formal analysis. **Ognjen Milicevic:** software, formal analysis. **Aleksa Palibrk:** data curation, formal analysis, methodology, visualization, validation. **Ivana Basta:** supervision. **Stojan Peric:** project administration. **Aleksandra Pavlovic:** supervision. **Zorica Stevic:** supervision. **Milija Mijajlovic:** writing – review and editing, project administration, supervision, resources, funding acquisition.

## Conflicts of Interest

The authors declare no conflicts of interest.

## Data Availability

The data that support the findings of this study are available from the corresponding author upon reasonable request.
